# Supplementary material for: Electricity from methane by reversing methanogenesis
Source: Nat Commun. 2017 May 17;8:15419. doi: 10.1038/ncomms15419 (PMC5442358; doi:10.1038/ncomms15419)
Supplement: Supplementary Information — Supplementary Figures, Supplementary Tables, Supplementary Note and Supplementary References [file ncomms15419-s1.pdf]

**Supplementary Figures**

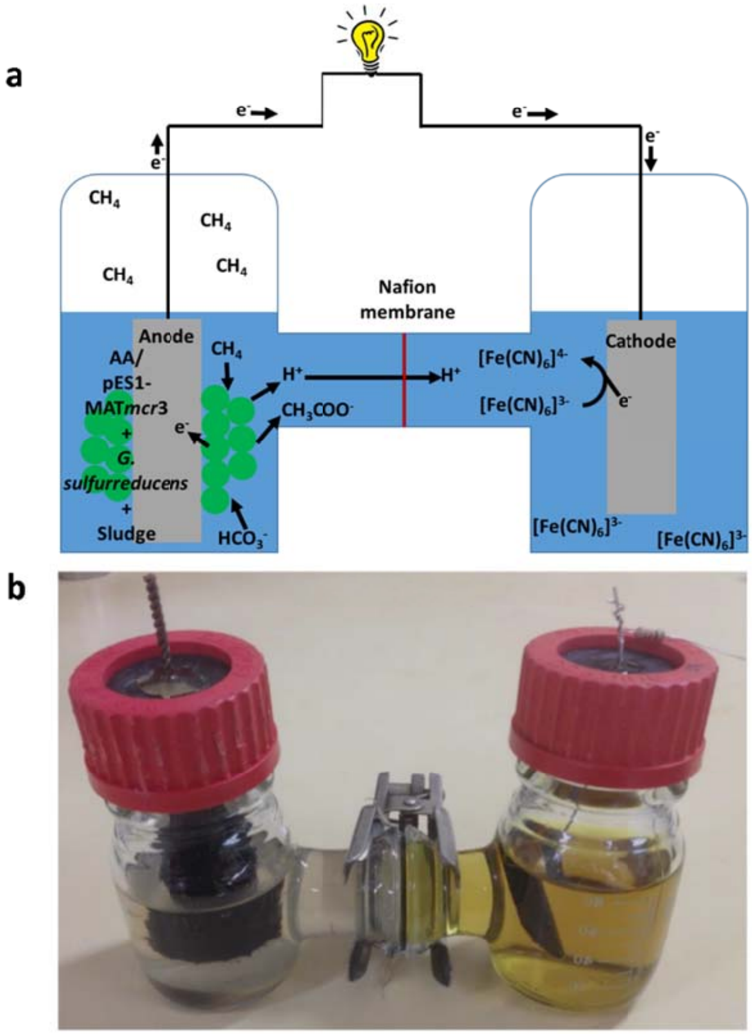


**Supplementary Figure 1. MFC with methane as the main carbon source.** (**a**) Schematic showing air-adapted *M. acetivorans* containing pES1-MAT*mcr*3 (AA/pES1-MATmcr3), *G. sulfurreducens*, and sludge under a methane headspace in the anode compartment. The cathode compartment includes a ferricyanide solution as the catholyte. (**b**) Picture of the actual MFC showing the carbon fiber brush electrode anode on the left and the carbon cloth cathode in yellow ferricyanide solution on the right.

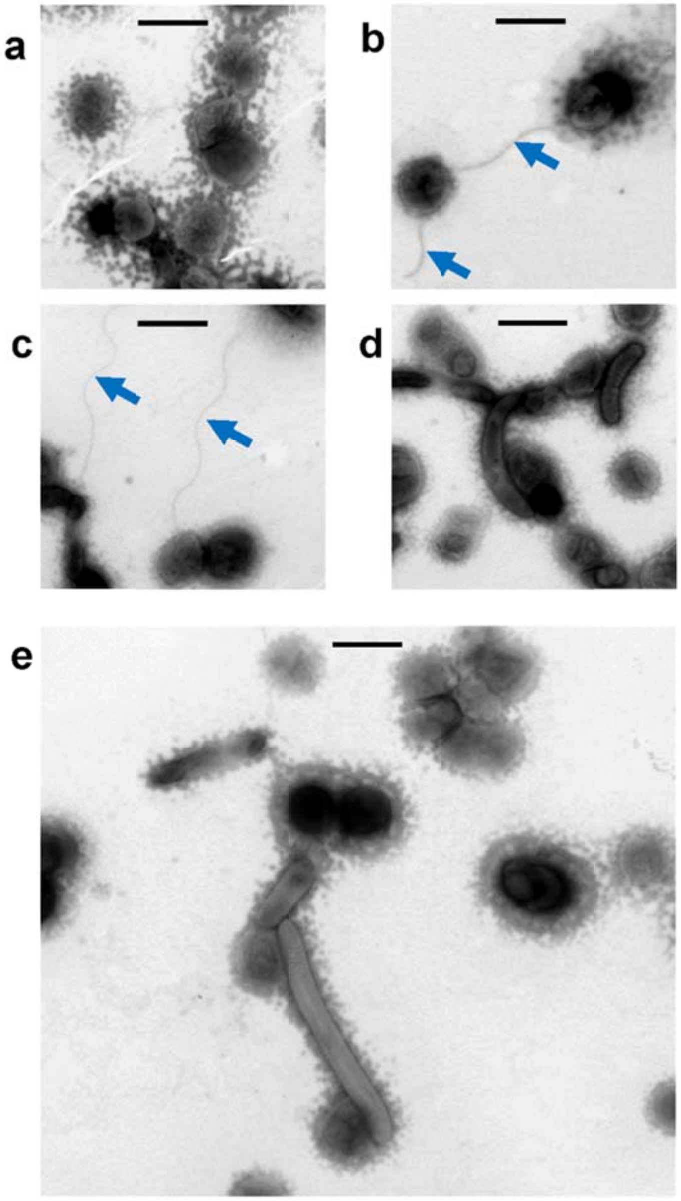


**Supplementary Figure 2. TEM images of the sludge inoculum.** Electron micrographs of sludge after two acclimations with methane as the carbon source with the terminal electron acceptor: 1 µM $FeSO_4$ (**a-c**), and 1 mM $FeCl_3$ (**d-e**). Scale bar is 1 µm. Blue arrows show pili.

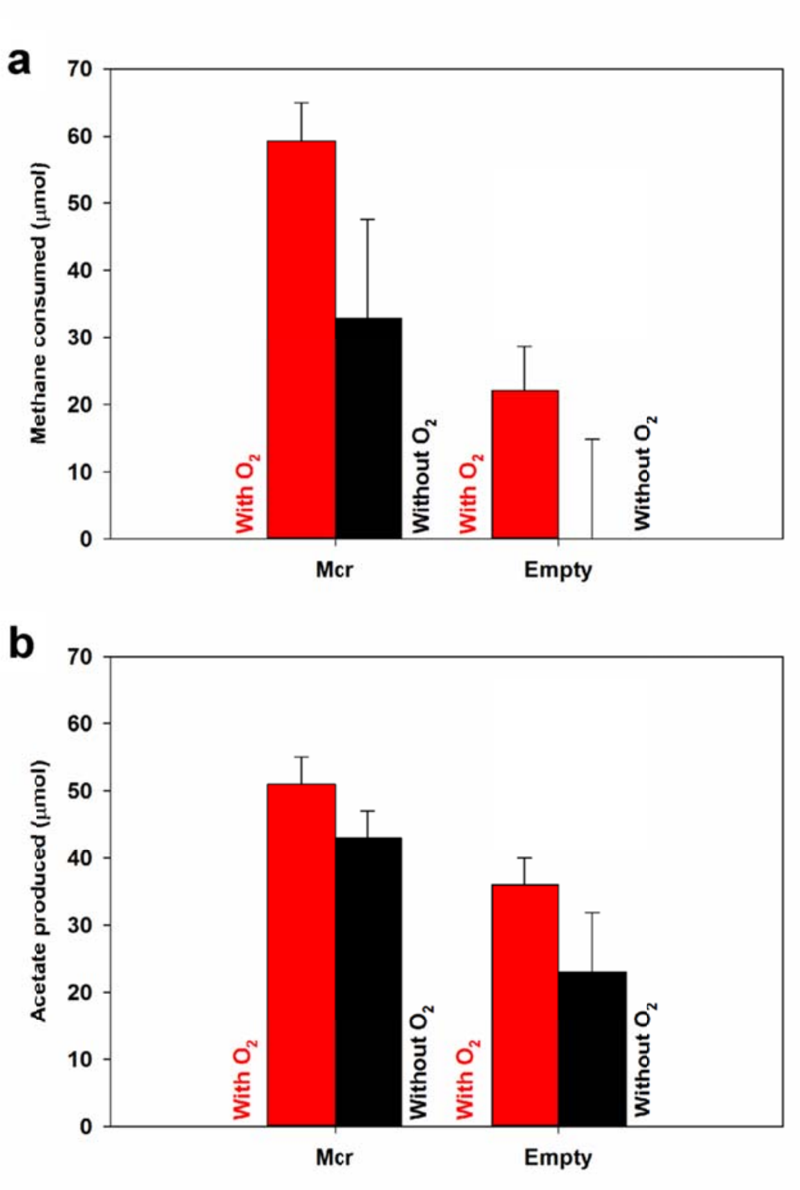


**Supplementary Figure 3. The air-adapted strain producing Mcr from ANME consumes methane and produces acetate in the presence of oxygen.** Cells of the air-adapted *M. acetivorans* strain containing pES1-MAT*mcr*3 ("Mcr") or pES1(Pmat) ("Empty") were placed at high cell-density under a headspace of pure methane or methane and 1% oxygen. The amounts of (**a**) methane consumed and (**b**) acetate produced were quantified, with averages and standard deviations between three replicates shown.

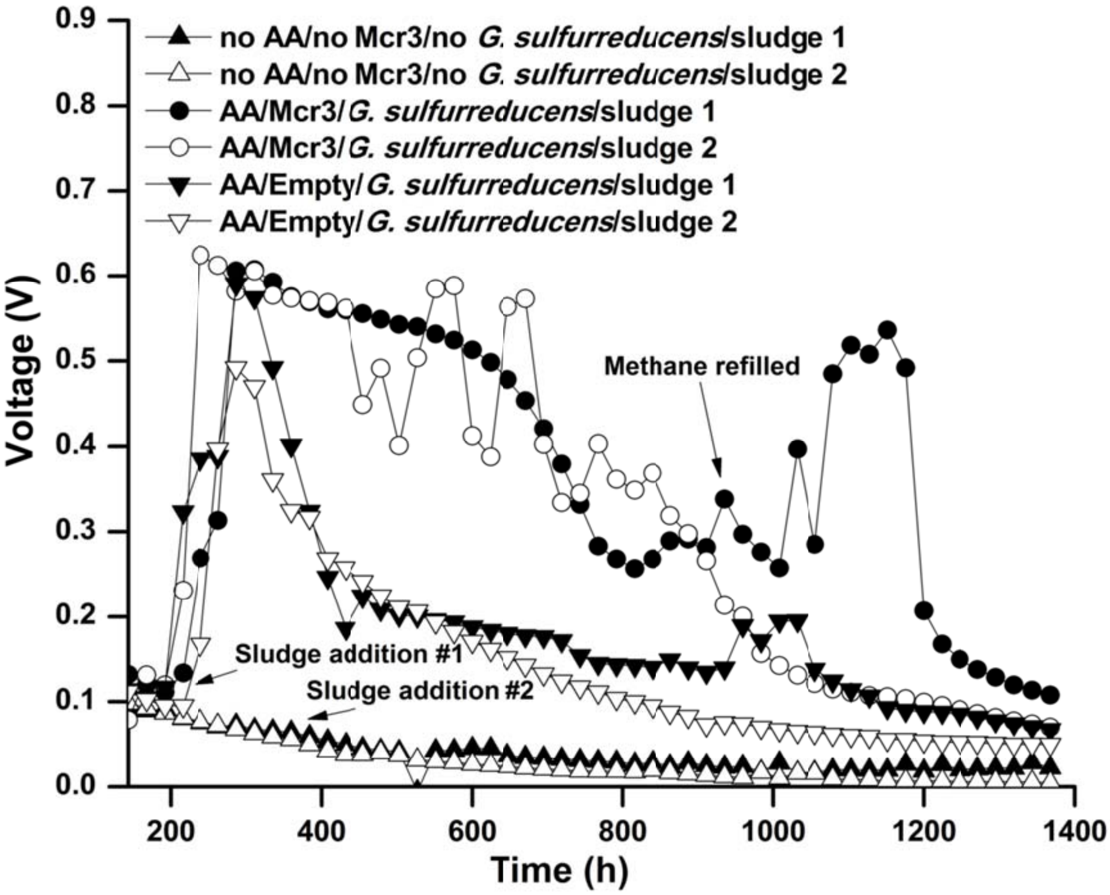


**Supplementary Figure 4. Time course of voltages generated in individual MFCs.** The MFCs contain methane in the headspace and include the air-adapted *M. acetivorans* host containing pES1-MAT*mcr*3 ("AA/Mcr3"), the air-adapted *M. acetivorans* host containing the empty plasmid pES1(Pmat) ("AA/Empty"), no *M. acetivorans* strains ("no AA/no Mcr3"), and *G. sulfurreducens,* as indicated. Sludge was added to the indicated MFCs after 6 d (sludge addition #1 to AA/Mcr3/*G. sulfurreducens*/sludge and AA/Empty/*G. sulfurreducens*/sludge MFCs) or after 15 d (sludge addition #2 to no AA/no Mcr3/no *G. sulfurreducens*/sludge). Methane consumption was quantified after 40 d, and replenished at that time.

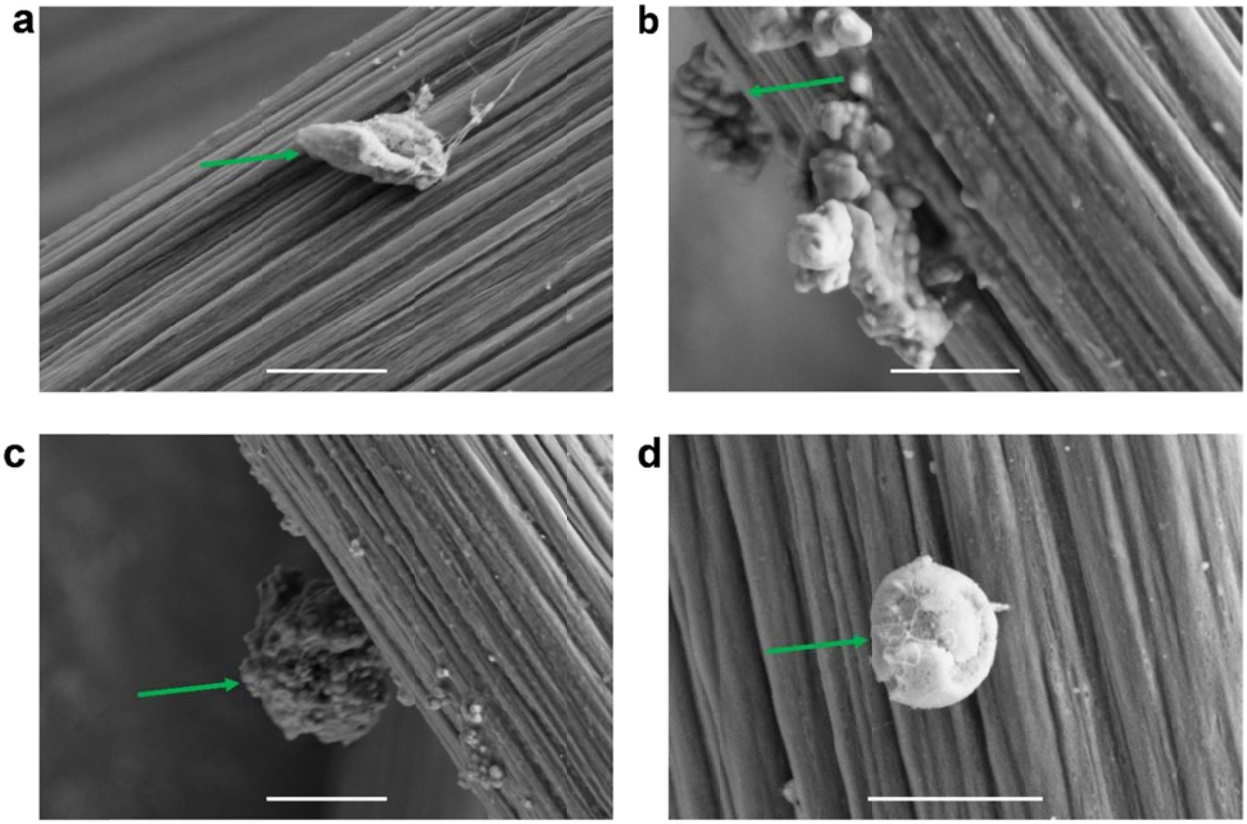


**Supplementary Figure 5. SEM visualization of cells attached to anodes.** Electron micrographs of anode carbon fibers from (**a**) an MFC inoculated with air-adapted *M. acetivorans* containing pES1-MAT*mcr*3, and (**b-d**) an MFC inoculated with the air-adapted *M. acetivorans* containing pES1-MAT*mcr*3, *G. sulfurreducens*, and methane-acclimated sludge. Scale bar is 1 µm. Green arrows show cells.

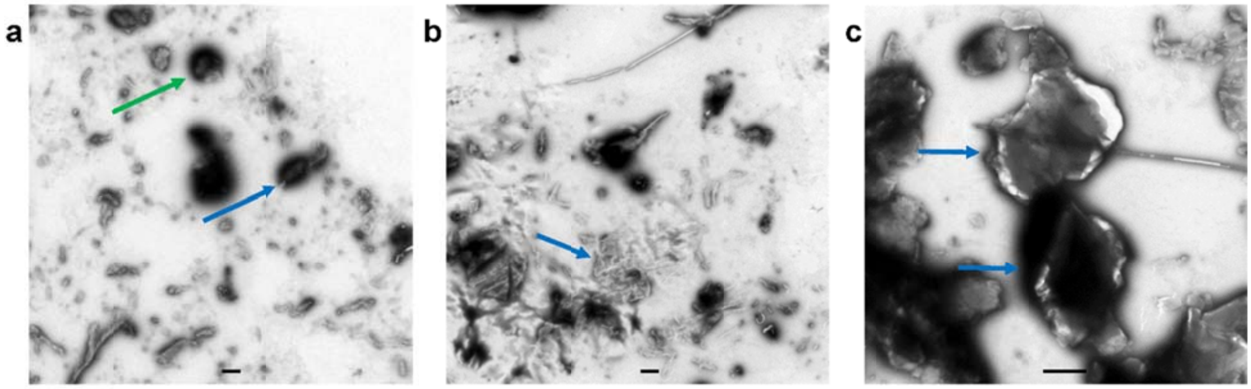


**Supplementary Figure 6. $FeCl_3$ crystal morphology**. (**a-c**) TEM visualization of salt crystals and *M. acetivorans* C2A containing pES1-MAT*mcr*3 grown in HS medium with methane and 10 mM $FeCl_3$. Scale bar is 1 µm. Blue arrows show salt crystals, and green arrows show putative cells.

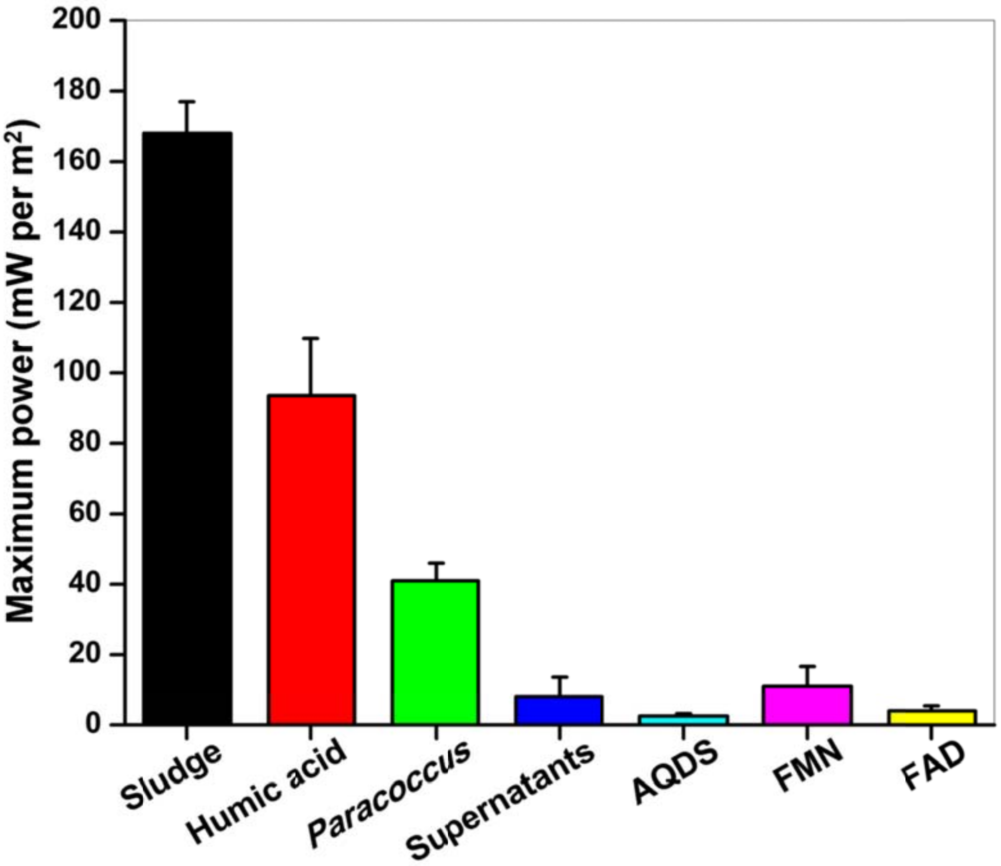


**Supplementary Figure 7. Maximum power generation in MFCs with sludge or exogenous electron shuttles.** Maximum power generated in each MFC is normalized by the cathode surface area of 0.00227 $m^2$. All MFCs contain methane in the headspace, air-adapted *M. acetivorans* with pES1-MAT*mcr*3, and *G. sulfurreducens*. Sludge, *P. denitrificans* DSM413, or an electron shuttle to replace the sludge (0.5 mM FMN, 0.5 mM FAD, 5 mM AQDS, 0.5% humic acids, and supernatants from methane-acclimated sludge) was added once the voltage of each MFC decreased to below a threshold value of 150 mV. Averages and standard deviations are shown.

## Supplementary Tables

**Supplementary Table 1. History of each of the 7 sludge cultures acclimated to methane.** All inoculations were done with 50 µL into 5 mL of medium, with all cultures kept in HS-methane (unless a different headspace is indicated) with different terminal electron acceptors (TEAs). Cultures with the same TEA mentioned in third round cultures 1 and 2 are the same culture (not different replicates), and all cell lines came from the same original sludge isolate. All cultures were incubated at 37°C without shaking.

| Third round culture | First round TEA | Second round TEA | Third round TEA |
|---|---|---|---|
| 1 | 1 mM $FeCl_3$ | 1 mM $FeCl_3$ | 1 mM $FeCl_3$ |
| 2 | 1 mM $FeCl_3$ | 1 mM $FeCl_3$ | 1 mM $FeCl_3$, headspace of 80% $CH_4$ 20% $CO_2$ |
| 3 | 1 mM $FeCl_3$ | 1 mM $FeCl_3$ | 0.001 mM $FeSO_4$ |
| 4 | 1 mM $FeCl_3$ | 1 mM $FeCl_3$ | 0.001 mM $FeCl_3$ |
| 5 | 0.001 mM $FeSO_4$ | 0.001 mM $FeSO_4$ | 40 mM ferrihydrite |
| 6 | 0.001 mM $FeSO_4$ | 0.001 mM $FeSO_4$ | 1 mM $FeCl_3$ |
| 7 | 0.001 mM $FeSO_4$ | 0.001 mM $FeSO_4$ | 0.001 mM $FeSO_4$, headspace of 95% $CH_4$ 5% $H_2$ |

**Supplementary Table 2. Strains and plasmids used in this study.**

| Strain | Description | Source |
|---|---|---|
| *M. acetivorans* AA | air-adapted *M. acetivorans* | [1] |
| *G. sulfurreducens* PCA | wildtype *G. sulfurreducens* | J. G. Ferry |
| *M. acetivorans* C2A | wildtype *M. acetivorans* | [2] |
| **Plasmids** | | |
| pES1(Pmat) | $Amp^R$, $Pur^R$, R6K *ori*, C2A *ori*, $P_{mcr\_ANME\text{-}1}$ | [3] |
| pES1-MAT*mcr*3 | $Amp^R$, $Pur^R$, R6K *ori*, C2A *ori*, $P_{mcr\_ANME\text{-}1}$::$mcr_{ANME\text{-}1}$ | [3] |

**Supplementary Table 3. Methane consumption after 40 days**. Methane loss from the no AA/no Mcr3/no *G. sulfurreducens*/sludge MFCs was subtracted from each MFC to determine the total methane consumption. Averages and standard deviations between three replicates are shown. All MFCs included methane in the headspace. MFCs included at time 0 the following as indicated: the air-adapted *M. acetivorans* host containing pES1-MAT*mcr*3 ("AA/Mcr3"), the air-adapted *M. acetivorans* host containing pES1(Pmat) ("AA/Empty"), *G. sulfurreducens*, and sludge. Sludge was added to the MFCs once the voltage of each indicated MFC decreased to a threshold value of 150 mV or below (after 6 or 15 d for AA/Mcr3/*G. sulfurreducens*/sludge and AA/Empty/*G. sulfurreducens*/sludge, or after 15 d for no AA/no Mcr3/no *G. sulfurreducens*/sludge). Methane amounts were measured in the anode compartments of all MFCs after 40 d.

| **MFC** | **Methane consumed (µmol)** |
|---|---|
| AA/Mcr3/*G. sulfurreducens*/sludge | 260 ± 40 |
| AA/Empty/*G. sulfurreducens*/sludge | 130 ± 40 |
| AA/Mcr3/no *G. sulfurreducens*/no sludge | 50 ± 50 |
| AA/Empty/no *G. sulfurreducens*/no sludge | 0 ± 100 |

**Supplementary Table 4. Oligonucleotides used in this study.** “f” indicates forward primer and “r” indicates reverse primer. Underlined sequences represent Illumina 5’ adapters. Degenerate nucleotides are denoted according to the International Union of Pure and Applied Chemistry where N represents A, C, T, or G; B represents C, G, or T; S represents G or C; and V represents A,  C, or G.

| **Primer name** | **Sequence (5’→3’)** |
|---|---|
| PRO341-f | <u>TCGTCGGCAGCGTCAGATGTGTATAAGAGACAG</u>CCTACGGGNBGCASCAG |
| PRO801-r | <u>GTCTCGTGGGCTCGGAGATGTGTATAAGAGACAG</u>GACTACNVGGGTATCTAATCC |

**Supplementary Note 1**

**Sample Calculation of Coulombic Efficiency**

Coulombic efficiency was calculated according to equation (1). For MFCs inoculated with air-adapted *M. acetivorans* containing pES1-MAT*mcr*3, *G. sulfurreducens*, and methane-acclimated sludge, under a methane headspace, the calculation is shown as equation (4).

$$CE = \frac{\left((0.38 \pm 0.04) - (0.046 \pm 0.004) - (0.063 \pm 0.002)\right) mA * (35\ d) * (86400\ s/d)}{8 \frac{mol\ e^{-}}{mol\ CH_4} * (1.197 \pm 0.002) mmol\ CH_4 * 96485 \frac{s * mA}{mmol}} * 100\% = 90 \pm 10\% \quad (4)$$

**Supplementary References**


1. Jasso-Chavez, R. *et al.* Air-adapted *Methanosarcina acetivorans* shows high methane production and develops resistance against oxygen stress. *Plos One* **10** (2015).
2. Sowers, K.R., Baron, S.F. & Ferry, J.G. *Methanosarcina acetivorans* sp. nov., an acetotrophic methane-producing bacterium isolated from marine sediments. *Appl. Environ. Microbiol.* **47**, 971-978 (1984).
3. Soo, V.W. *et al.* Reversing methanogenesis to capture methane for liquid biofuel precursors. *Microb. Cell Fact.* **15**, 11 (2016).
